# A View of the Marriage Question

**Published:** 1889-09

**Authors:** 


### A VIEW OF THF MARRIAGE QUESTION.

Nine-tenths of the unhappy marriages are the result of green human calves being allowed to run at large in society pastures without any yoke on them. They marry and have children before they do mustaches. They are fathers of twins before they are proprietors of two pairs of pants, and the little girls they marry are old women before they are twenty years old.

Occasionally one of these gosling marriages turns out all right, but it is a clear case of luck.

If there was a law against young galoots sparking and marrying before they have cut all their teeth, we suppose the little cusses would evade it in some way. But there ought to be a sentiment against it.

It is time for these bantams to think of finding a pullet when they have raised money enough to buy a bundle of lath to build a hen-house. But they see a girl who looks cunning, and they are afraid there is not going to be enough to go round, and then they begin to spark real spry, and before they are aware of the sanctity of the marriage relation, they are hitched for life, and before they own a cook stove or a bedstead they have got to get up in the night and go after the doctor, so frightened that they run themselves out of breath and abuse the doctor because he does not run, too. And when the doctor gets there, there is not linen enough in the house to wrap up the "baby."

